# Realized niche and microhabitat selection of the eastern green lizard (*Lacerta viridis*) at the core and periphery of its distribution range

**DOI:** 10.1002/ece3.4612

**Published:** 2018-10-31

**Authors:** Ana M. Prieto‐Ramirez, Guy Pe’er, Dennis Rödder, Klaus Henle

**Affiliations:** ^1^ Department of Conservation Biology Helmholtz Center for Environmental Research UFZ Leipzig Germany; ^2^ Department of Herpetology Zoological Research Museum Alexander König ZFMK Bonn Germany; ^3^ Department of Economics and Department of Ecosystem Services UFZ Leipzig Leipzig Germany; ^4^ German Center for Integrative Biodiversity (iDiv) Halle‐Jena‐Leipzig Leipzig Germany; ^5^ Universität Leipzig Leipzig Germany

**Keywords:** *Lacerta viridis*, mixed models, niche differentiation, niche hypervolume, peripheral populations, spatial autocorrelation

## Abstract

The available range of habitats and suitable abiotic conditions like temperature and radiation tends to be narrower toward the periphery of the distribution range of species. Peripheral populations of generalist species could then be more specialized and have a smaller and differentiated realized niche (habitat niche in our study) compared to populations at the core. Likewise, patterns of microhabitat selection can differ between periphery and core. In our study, we compared niche size and microhabitat selection among core (Bulgaria) and northern peripheral (Germany, Czech Republic) populations of *Lacerta viridis* and estimated niche differentiation among regions. We collected data on vegetation structure and abiotic parameters at the microhabitat scale in each region. In order to compare niche size among regions and estimate niche differentiation, we built multidimensional niche hypervolumes. We applied generalized linear mixed models and model averaging, accounting for spatial autocorrelation when necessary, to analyze microhabitat differences among regions and microhabitat selection in each region. Peripheral populations were more specialized, having a smaller niche than core ones, and their niche differed from that in the core (S**ø**rensen overlap in all comparisons <0.3). Microhabitats at the periphery had lower radiation and soil compaction and less structured vegetation. Microhabitat selection at the core depended solely on abiotic parameters, while at the periphery it was defined by only vegetation structure (Czech Republic) or a combination of both, vegetation structure, and abiotic factors (Germany). Thus, peripheral populations seem to compensate for overall harsher climatic conditions by responding to different parameters of the microhabitat compared to core populations. We suggest specific conservation measures for *L. virids* in each studied region and point out the general implications of a higher specialization degree of peripheral populations in relation to climate change and habitat fragmentation.

## 1 INTRODUCTION

Availability of resources and environmental conditions changes along the distribution range of species, with especially marked differences along the gradients of broadly distributed species (Gaston, 2009; Kirkpatrick & Barton, 1997). These patterns can lead to ecological differences between populations of the same species living either at the core or at the periphery of its distribution range (Brown, Stevens, & Kaufman, 1996). The Kühnelt principle (Kühnelt, 1965) states that the range of colonizable habitats is wider at the core where environmental conditions are optimal, whereas at the periphery conditions are suboptimal and fewer microhabitats are suitable for the species. Therefore, populations at the core should be habitat generalists (“euryoecious”), while populations at the periphery of the species’ range can, in comparison, be more specialists (“stenoecious”) (Böhme & Rödder, 2014). Under the Hutchinson's concept of ecological niche (Hutchinson, 1957), this suggests that populations living at the periphery of the distribution range will have a smaller locally realized niche breadth compared to generalist core populations. Studies quantifying these differences in animal populations are scarce, but evidence of smaller niche breadth at the periphery compared to the core has been found in a few taxa. For instance, the niche breadth and availability of resources of three invertebrate species, the butterfly *Plebejus argus*, the ant *Myrmica sabuleti*, and the grasshopper *Chorthippus vagans*, were found to decrease toward the northern colder edge of their distribution range (Thomas, Rose, Clarke, Thomas, & Webb, 1999). In vertebrate species, Lappalainen and Soininen (2006) found that the niche breadth of fresh water percid and cyprinid fishes was narrower toward the northern edge of the distribution range, and Yurkowski et al. (2016) demonstrated that niche breadth at the population level decreased with increasing latitude in ringed seals (*Pusa hispida*) and beluga whales (*Delphinapterus leucas*).

Additional to differences in niche breadth, niche differentiation can also be found when comparing core and peripheral populations. Studies investigating niche differentiation in animal species are focused on evolutionary niche divergence among populations across the species’ distribution range (Ahmadzadeh et al., 2013; Cadena & Loiselle, 2007), with the niche of relict populations being usually found to be differentiated from that of more central populations (Lozano‐Jaramillo, Rico‐Guevara, & Cadena, 2014). Many approaches exist for such studies, such as occupancy models with climatic, land cover, or other environmental variables as covariates (Araújo & Peterson, 2012; Chefaoui, Hortal, & Lobo, 2005; Hirzel & Le Lay, 2008), and models that use presence/pseudoabsence data (Morales, Fernández, Carrasco, & Orchard, 2015). These studies are generally done at a macroscale of large regions (often including the whole distribution of a species) and using a coarse spatial resolutions of 1 km^2^ or more (Pearson & Dawson, 2003). Such studies are unable to assess the effects of environmental factors that have a much finer spatial variability. There is a lack of studies on animal species testing niche differentiation by using field data at such microhabitat scale that allows deeper insights into intraspecific niche differences between peripheral and core populations, and into the microhabitat selection patterns shaping these differences. Elucidating such differences is important for understanding ecological processes like range shifts under global change, as well as for promoting effective conservation measures for edge populations of threatened species (Lesica & Allendorf, 1995; Peterman, Feist, Semlitsch, & Eggert, 2013).

Given their sensitivity to environmental changes and thermal dependency, reptiles are of particular interest to study niche and microhabitat selection in regions with different ranges of available habitats and climatological regimes (Buckley, 2010; Cunningham, Rissler, Buckley, & Urban, 2016). Moreover, for some taxa like lacertid lizards, there is enough qualitative information about niche differences between core and peripheral populations, like the known differences in the diversity of habitats occupied in core regions of the distribution range compared with the northern periphery (Korsós, 1982; Olsson, 1988). *Lacerta viridis*, for example, is a common species in the Balkan Peninsula in Eastern Europe and Asia Minor (Elbing, 2001) and has its northern distribution range located in Germany and in the Bohemian region of the Czech Republic. In core regions, the species is found in habitats ranging from slopes with rock covering, bushlands, and road edges to mixed forest and pine plantations, including several semi‐natural and urban habitats (Heltai, Sály, Kovács, & Kiss, ; Covaciu‐Marcov et al., 2009; Popgeorgiev & Mollov, 2005). In Germany and Czech Republic, where thermal conditions and other limiting factors like daily hours of sunshine (Frör, 1986; Laube & Leppelsack, 2007) do not provide many suitable habitats for the species, it is scarce and mostly found in open areas and river valleys (Böhme & Moravec, 2011; Böhme, Schneeweiß, Fritz, Schlegel, & Berendonk, 2007). However, despite substantial descriptive evidence suggesting a narrower range of habitats used by northern edge populations, there are no quantitative studies that explicitly quantify and compare the niche between core and peripheral populations, nor any study comparing the factors that determine microhabitat selection in different regions.

**Figure 1:**
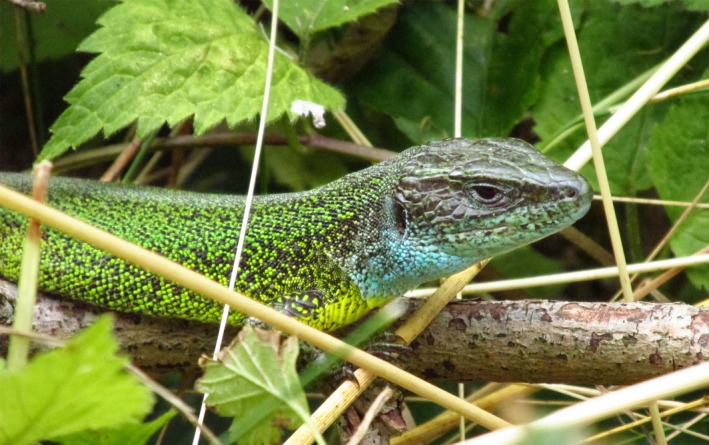
Adult male of the **e**astern green lizard *Lacerta viridis* in Passau, Germany. Photo credits: AMPR

In the present study, we compare the specialization degree with respect to realized niche, and microhabitat selection of populations of *L. viridis* (Figure 1) living either at the core (Bulgaria) or at the northern periphery (Germany and Czech Republic) of the species’ distribution range (Figure 1).The studied populations in the Czech Republic are relict populations, which are not part of the continuous distribution of the species, and in Germany and the Czech Republic, the species is critically endangered and highly protected according to the EU Habitats Directive and national conservation regulations..On the other hand, in Bulgaria, *L. viridis* is the most common lizard species (Beutler & Rudolph, 2003; Zavadil & Moravec, 2003). We expected to find (a) smaller realized niches in northern edge populations compared to the core, with a niche differentiation present in populations located around Prague (relicts) but not in those in Passau (which are part of the continuous distribution range); (b) higher preference of *L. viridis* in the periphery for specific vegetation structures at the microhabitat scale, like low and open vegetation, as compensation for overall suboptimal climatic conditions; and (c) higher influence of vegetation structure in the microhabitat selection in the northern periphery, where the availability of suitable habitats for the species is a limiting factor, while in the core, where the available range of habitats is broader, abiotic parameters will have a higher influence in the microhabitat selection.

## 2 MATERIALS AND METHODS

### 2.1 Study regions and site selection

**Figure 2:**
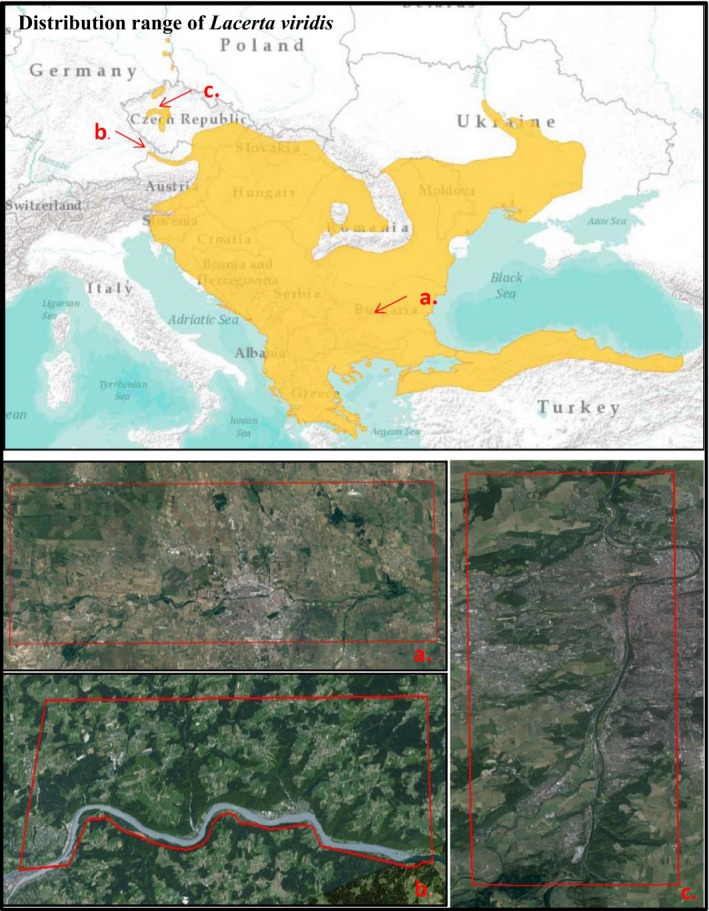
Distribution range of *Lacerta viridis* and study sites in the core located in Plovdiv, Bulagria (a), and in two peripheral regions corresponding to Passsau, Germany (b) and Prague, Czech Republic (c)

The study region at the core of the species’ distribution was located in the Thracian Plain of Bulgaria, in the surroundings of Plovdiv (Figure 2a). Bulgaria is the historical and current range core of the species (Popgeorgiev & Mollov, 2005),and in the Thracian Plain are represented most of the habitats in which *L. viridis* is present in central regions, from road edges and open shrublands to mesophilic forest. The study regions at the species’ northern periphery were located near Passau (Bavaria, Germany) and in the surroundings of Prague (Bohemia, Czech Republic). From now on, we will use the term *periphery* to refer to the study regions located in the northern periphery. In Passau (Figure 2b), populations are found along the Danube Valley in rocky outcrops in the oak and hornbeam forest and on the southern exposed cliffs, but mostly along an abandoned railroad that runs parallel to the river. Populations of *L. viridis* in the surroundings of Prague (Figure 2c) are relict populations located in open stony areas of the oak forest and on the slopes of the Moldova valley, as well as those of other valleys perpendicular to the Moldova River.

The extent of the areas where the study was carried out in each region was 325 km^2^, 288 km^2^, and 522 km^2^ in Plovdiv, Passau, and Prague, respectively. Based on information available about places where the species has been found and on information about the habitat of *L. viridis *reported in the literature, we identified potential suitable sites into these areas by using satellite maps. Each site represented a portion of habitat potentially holding a population and separated from other sites/populations by structures in the landscape (e.g., agriculture, highways) that do not represent habitat. In order to reduce the effects of probable local processes present in each region, we increased as much as possible the number of sites, by visiting all potentially suitable sites present in the study area in each region. In total, we visited 40, 27 and 33 sites visited in Plovdiv, Passau, and Prague, respectively. Also, to avoid bias in the habitat types visited in each region, at the periphery, we also visited sites with similar vegetation structure to those where *L. viridis* was found in the core (e.g., mixed forest). In Plovdiv, the area of the sites was 0.1–3.91 km^2^ and the distance between sites was 5–6,100 m; in Passau, sites had an area of 0.23–4.51 km^2^ and were apart from one another 10–800 m; in Prague, sites were 0.3–2.28 km^2^ large and the range of distances between was 5–2,171 m.

### 2.2 Field survey and data collection

Field surveys took place in Plovdiv and Passau in 2014 and in Prague in 2015. In order to make the surveys comparable among regions, they were carried out in each region starting with the onset of the reproduction season: early April in Plovdiv and early to mid May in the two peripheral regions. Sampling lasted till late May in Plovdiv (core) and till June and July in Passau and Prague. This shift in sampling made average maximum air temperatures per sampling month similar among sites: 18.5 and 23.4°C in Plovidiv, 23.1 and 24.8°C in Passau, and 22.5 and 24.6°C in Prague).

Data were gathered around a total of 363 points, from which 152 were in the core (presence: 102; absence: 50), 117 in the periphery‐Pa (33; 84), and 94 in the periphery‐Pr (29; 65). In the core region, lizards were found in a variety of habitats from shrublands to mixed forest, in riverbeds as well as far away from any water body. In Passau, the presence of the lizards is restricted to the lower part of the narrow Danube valley, where the habitat is represented by stony areas with low vegetation. Finally, in Prague, lizards were mainly found in the open rocky slopes of the Vltava valley and the valleys of tributary rivers.

We used an occupancy survey design to incorporate detection probability. Following study designs proposed by Mackenzie and Royle (2005) and based on estimates of detection probability for similar species (Janssen & Zuiderwijk, 2006; Sewell, Guillera‐Arroita, Griffiths, & Beebee, 2012), the number of visits per site was set to two, one in the morning (9:00–12:00 a.m.) and one in the afternoon (14:00–19:00 p.m.) in accordance with the species’ daily activity pattern (Korsós, 1983). The second visit in each population was carried out either on the same day or one day later. Only in two populations in Plovdiv (core) and two in Prague visits were separated by 7 days.

Each visit lasted one hour, and sites were surveyed by means of line transects. Walking speed was standardized at 20 m/min. Thus, one hour visit corresponded to 1,200 m, which were divided into transects of variable lengths (50–400 m). Transects were systematically placed in order to represent the area of the site and all different habitat types present at it. With the use of maps and based on the relative coverage of each habitat type into each site, we calculated the length of each transect and the number of transects that had to be placed in each habitat type. The entire length of each transect was placed only in one habitat type and did not crossed to another. The number of transects surveyed per site ranged from 3 to 12. To avoid double counting of observed lizards among transects, the minimum distance between transects was 100 m. A width of 2.5 m at each side of the transect was set to carefully inspect visually for *L. viridis*. A metal stake was placed on the specific point where each lizard was seen and coordinates were taken. In a 25‐m^2^ plot around this point (presence plots), data on vegetation structure and abiotic parameters were recorded. Percentage of vegetation coverage was visually estimated for the following categories: herbs with a height lower than 30 cm (herbs1), between 40 and 80 cm (herbs2) and higher than 90 cm (herbs3); woody plants < 2 m and woody plants > 2 m; dry leaves, rocks and fallen trunks (rocks_trunks), bare soil, way (road edges, dirt tracks, walking paths), and coverage of branches (Branches). Vegetation height was measured with a retractable measure tape. Abiotic parameters included air temperature at 1.5 m height, 10 cm height and ground surface, soil compaction, soil composition, slope, and aspect. Temperatures and soil compaction were measured at three random points (different for each parameter) within each plot and then averaged for the analysis. Soil compaction was measured with a manual penetrometer, and soil composition was qualitatively classified into humus, organic, clay, gravel, or sand. Temperature was measured with a precision digital thermometer (Greisinger GTH 175/PT), exposition was taken with a GPS (Garmin 62S) and slope with a compass (Global system DS 50G).

In order to analyze microhabitat preference of the species, the same data were collected in 25‐m^2^ plots around random points along each transect, where the lizard was not seen at the time of the survey. These random plots are specific locations that the lizard might use at other time and where it might not be permanently absent, but in order to simplify terminology, from now on we will call them absent plots. Random points were chosen by blindly selecting points along each transect in the GPS. Data gathering in each presence/absence plot took approximately 15 min, which were not accounted for as sampling time, and in consequence one hour of surveying lizards represented 2–4 hr of data sampling. Therefore, due to time constrains, data were gathered around a maximum of three “presence” points per transect per visit in the case more than three lizards were encountered, and a minimum of one random “absence” point per transect. If a lizard was encountered in an already surveyed plot during the second visit, data were not included in the analysis to avoid pseudo‐replication.

Additionally, to variables measured in the field, we estimated radiation at each data point and at the specific time range of the study in each region with the “*Potential incoming solar radiation”* tool of the software SAGA. For this purpose, elevation maps with 30‐m resolution were obtained from the USGS database. Aspect was transformed into two variables: cosine values, representing the South‐North component (S‐N aspect), and sine values, representing the West‐East component (W‐E aspect). S‐N aspect values increase from south to north, and W‐E aspect values increase from west to east.

### 2.3 Data preparation and variable selection

The following procedure was performed for the data set including all regions (see section Comparison of microhabitats among regions), and separately for the individual dataset of each region (see section Microhabitat selection in each region).Vegetation structure data were ARCSIN transformed, tested for correlation with Spearman rank correlation, and assessed for collinearity by estimating the variance inflation factor (VIF). Variables with correlation > 0.6 or VIF > 3 were excluded from analysis (Zuur, Ieno, & Elphick, 2010). In the dataset, including all regions, no correlation or collinearity was found and all variables were retained (Supporting Information Appendix S1, Table S1.1). In Plovdiv, the variable Herbs 2 had a high collinearity (VIF = 17) and was excluded from the analysis of microhabitat selection (see analysis description below). In the other two regions, neither correlation nor collinearity was found (Supporting Information Appendix S1, Table S1.2–S1.4). Therefore, all variables were retained. Continuous abiotic variables were log‐transformed and tested for correlation with the Pearson correlation test and also for collinearity with VIF. Variables with correlation >0.6 or VIF > 3 were excluded. Air temperature, temperature at 10 cm height, and temperature at soil surface were correlated (*r* > 0.9) in all study regions; hence for further analysis, only the temperature at the soil surface was used, as lizards’ bodies are directly in contact with it, and its influence on microhabitats may be the strongest. No correlation or collinearity was found in other variables (Supporting Information Appendix S1, Table S1.1–S1.4). Correlations between each abiotic continuous variable and the factor soil composition were tested using linear regression. In Plovdiv, soil composition was correlated with soil compaction (*F*_4,136_ = 3.75, *p* < 0.01) and radiation (*F*_4,136_ = 10.08, *p* < 0.001) and therefore removed from the analysis. In Passau and Prague, soil composition was correlated with soil compaction (*F_2_*_,98_ = 3.14, *p* = 0.047; *F*_3,73_ = 4.45, *p* = 0,038). To select between soil compaction and soil composition, we tested the effect of each of the two variables on the presence/absence of the lizard in each region and retained the variable with the strongest effect (Poulin, Villard, Edman, Goulet, & Eriksson, 2008). In all regions, soil composition was least correlated with presence/absence of *L. viridis,* and therefore, for further analysis this variable was removed.

### 2.4 Statistical analysis

#### 2.4.1 Niche size and specialization

To compare realized niches among regions, multidimensional niche hypervolumes were derived with the package “Hypervolume” from R software (Blonder, 2015). All calculations were performed separately for vegetation structure and abiotic parameters in each region. Data were scaled and centered, and principal component analysis (PCA) with the R package “ade4” (Dray, Dufour, & Thioulouse, 2015) was applied to the whole dataset including all points of all regions. This reduction in dimensionality was necessary as the niche hypervolume analysis requires orthogonal axes. Principal components with eigenvalues > 1 were used to construct the hypervolumes of the realized niches in each study region (see Table 1 for variable loadings). Six principal components were selected for vegetation structure (77.05% of total variance) and three for abiotic parameters (62.89% of total variance). We used a fixed bandwidth of 0.5 with 1,000 Monte Carlo samples per data point to calculate the volumes. Hypervolume units are standard deviations (*SD*). Besides the size of each hypervolume, we also estimated the intersection and the union, and for testing niche differentiation, we estimated the Sørensen overlap index for each comparison, which is an index measuring the similarity among two samples with values varying from 0 for low overlap to 1 for complete overlap (Blonder, Lamanna, Violle, & Enquist, 2014).

**Table 1:** Loadings of each variable in the principal components with eigenvalues >1 selected to build the niche hypervolumes for vegetation structure and abiotic parameters

	Principal components					
	PC1	PC2	PC3	PC4	PC5	PC6
Vegetation structure						
Herbs 1	0.19	0.73	−0.37	−0.08	0.09	0.12
Herbs 2	−0.43	−0.32	0.16	−0.24	0.48	0.06
Herbs 3	−0.38	−0.07	−0.28	0.16	−0.38	−0.49
Woody plants <2 m	0.10	0.17	0.64	0.30	−0.14	0.38
Woody plants >2 m	0.20	0.01	0.17	−0.51	0.26	−0.15
Dry leaves	0.50	−0.46	−0.11	0.05	−0.14	0.07
Rocks_trunks	−0.08	−0.25	−0.35	0.46	0.12	0.43
Bare soil	0.17	0.07	0.37	0.41	0.09	−0.58
Way	−0.11	−0.06	0.15	−0.40	−0.70	0.17
Branches	0.53	−0.24	−0.17	−0.12	−0.01	−0.13
Abiotic parameters						
Temperature	−0.34	−0.09	0.72			
Soil compaction	0.37	0.35	0.53			
S‐N aspect	0.05	−0.81	0.13			
W‐E aspect	−0.25	0.06	−0.43			
Slope	−0.56	0.41	0.06			
Radiation	0.60	0.20	−0.06			

#### 2.4.2 Comparison of microhabitats among regions

For comparing microhabitats among regions, a multinomial logistic regression was run using the “*multinom”* function of the “nnet” R package (Ripley & Venables, 2016), with “region” as response variable. Analysis was first done separately for vegetation structure and abiotic parameters. After fitting a global model with all variables of either vegetation structure or abiotic parameters, all possible models with a reduced number of parameters were generated with the “*dredge”* function of the “MuMIn” R package (Bartón, 2015). Model comparison was based on Akaike's information criterion corrected for small sample size (AICc) (Burnham & Anderson, 2002). All models with ∆AICc<2 relative to the best model were selected, and parameters were estimated by averaging across these models with the “*model.avg”* function of “MuMIn” package. Relative variable importance (RVI) was calculated by summing the Akaike weights of each variable across the selected models. Variables with RVI > 0.6 were considered important (Kennedy et al., 2013). Important variables of both sets of variables, vegetation structure and abiotic parameters, were then combined in a third global model. Again, all possible models were generated and those with ∆AICc < 2 were averaged. We selected the approach of analyzing vegetation structure and abiotic parameters separately, and then combine most important variables of both averaged models in order to avoid overfitting of the global model, which is a common risk in mixed models that tends to overweight the variables averaged through the best models (Grueber, Nakagawa, Laws, & Jamieson, 2011).

#### 2.4.3 Microhabitat selection in each region

We applied generalized linear mixed models GLMM, with plot presence/absence as response variable, site occupancy (i.e., the presence or absence of the lizard in each visited site) as random factor and variables of vegetation structure or abiotic parameters as fixed factors. Analyses were initially done separately for vegetation structure and abiotic parameters. For each region, a full model containing all variables, either of vegetation structure or of abiotic parameters, was fitted using the “*glmer”* function of the “lme4” R package (Bates, Maechler, Bolker, & Walker, 2016) with a logit link function and binomial error distribution. We tested for spatial autocorrelation of residuals (SACR) and when present, we applied principle coordinates of neighbor matrices (PCNM) (See “Detection and correction of spatial autocorrelation”). We then proceeded as described in Comparison of microhabitats among regions to generate all possible models, averaged through those with ∆AICc < 2 and combine the most important variables of both the vegetation structure and abiotic parameters averaged models. We checked again for VIF and for SACR, and the process of model averaging was repeated to obtain the final model that includes the most important variables among vegetation structure and abiotic factors. For each final model, we report conditional R^2^ corresponding to the variance explained by fixed factors and random term together, and marginal *R* ^2^ representing the variance explained by fixed factors only (Nakagawa & Schielzeth, 2013).

#### 2.4.4 Detection and correction of spatial autocorrelation

All global models (vegetation structure, abiotic parameters, or combinations thereof) of microhabitat selection in each region were tested for spatial autocorrelation of model residuals (SACR) by estimating Moran's I index, calculating Moran's I‐based correlograms and computing autocorrelation of residuals. Correction for SACR was done by means of principal coordinates of neighbor matrices (PCNM). PCNM are a type of Moran's eigenvector maps and consist of calculating spatial eigenvectors based on a matrix of truncated distances. The obtained PCNM vectors can then be added into the model as fixed terms to account for SACR (Borcard & Legendre, 2002) (Supporting Information Appendix S2).

## 3 RESULTS

### 3.1 Niche size and specialization

**Figure 3:**
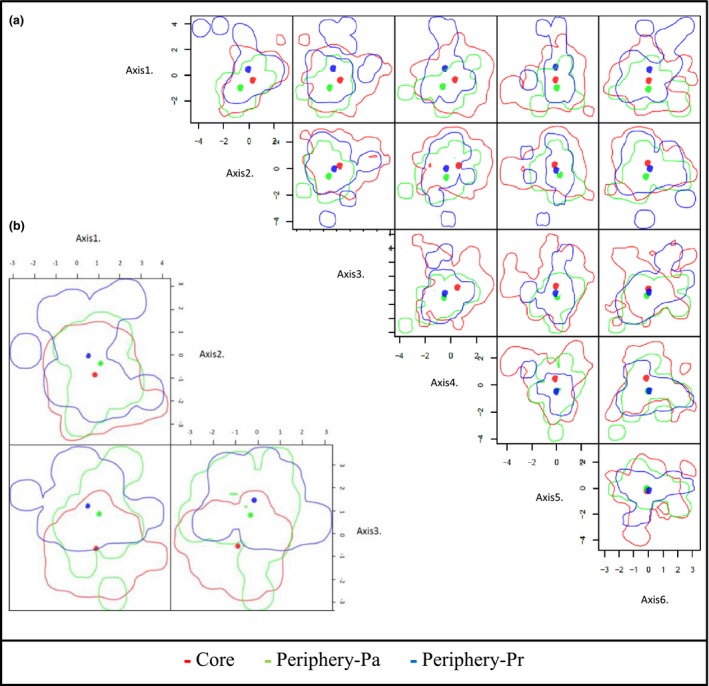
Two dimension (2D) representation of the multidimensional niche hypervolumes of realized niches for vegetation structure (a, 6 dimensions) and abiotic parameters (b, 3 dimensions) in the core of the distribution range of *L. viridis* (core, red), in the periphery in Passau (periphery‐Pa, green) and in the periphery in Prague (periphery‐Pr, blue). Dimensionality of each niche hypervolume corresponds to the number of principal components with eigenvalue >1

The realized niche of vegetation structure was largest in the core, followed by the periphery‐Pa and the periphery‐Pr (Table 2, Figure 3a). The realized niche of vegetation structure was found to differ in both peripheral regions from the niche in the core with the same degree of differentiation (Sørensen overlap = 0.1). Percentages of intersected niche volume ranged between 21.93%–23.18% for the peripheries and 6.5%–7.36% for the core. Between peripheral regions, there was also differentiation (Sørensen overlap = 0.08) and low percentages of overlapped niche volumes (8.24% for periphery‐Pa and 8.80% for periphery‐Pr).

**Table 2:** Comparison among the realized niche size in Plovdiv (Pl), Passau (Pa), and Prague (Pr)

Comparison	Volume 1	Volume 2	Intersection	Union	Sørensen overlap
Vegetation structure					
Pl–Pa	90.89	28.85	6.69	113.05	0.11
Pl–Pr	90.89	27.03	5.93	111.98	0.10
Pa–Pr	28.85	27.03	2.38	53.50	0.08
Abiotic parameters					
Pl–Pa	32.89	20.97	10.16	47.70	0.37
Pl–Pr	32.89	23.24	4.29	51.84	0.15
Pa–Pr	20.97	23.24	6.32	37.89	0.28

Note

Volume 1 and 2 correspond to the first and second region mentioned in the name of each comparison.

The realized niche based on abiotic parameters was also largest in the core, but in this case, it was followed by that in the periphery‐Pr and the smallest abiotic niche was in the periphery‐Pa (Table 2, Figure 3b). In both peripheral regions, it differed from that in the core, with the lowest overlap found between the Periphery‐Pr and the core (Sørensen overlap = 0.15), with 18.45% of the niche in periphery‐Pr intersecting with 13.04% of the niche in the core. Between periphery‐Pa and core (Sørensen overlap = 0.37) intersected volumes were 48.45% and 30.89%, respectively. The comparison between peripheries also showed niche differentiation (Sørensen overlap = 0.28), and 30.13% of the niche of Periphery‐Pa overlapped with 27.19% of the niche in Periphery‐Pr.

### 3.2 Comparison of microhabitats among regions

With the multinomial logistic regression (Table 3), we found that the most important variables differentiating microhabitats used among regions were radiation, soil compaction, Herbs1, Herbs2, Herbs3, woody plants<2 m, woody plants>2 m, and Way (RVI = 1). In both peripheral regions, radiation and soil compaction were lower compared to the core region. Also, herbs and woody plants had a lower proportion in microhabitats used in peripheral regions compared to the core region. When comparing between peripheral regions microhabitats used in periphery‐Pr had an even lower radiation and proportion of herbs and woody plants<2 m, but higher soil compaction and woody plants>2 m. Most of the populations in Prague were found on rocky slopes of the valley, with sparse vegetation and scarce trees. Given the rocky substrate of slopes inhabited by *L. viridis* in Prague, the soil compaction was higher in Prague compared to Passau (Supporting Information Appendix S3, Table S3.1 for model selection and model averaging separately for vegetation structure and abiotic parameters).

**Table 3:** Parameter estimates with 95% confidence interval (LCL and UCL) from averaged models of the multinomial logistic regression for the comparison among realized niches in Plovdiv (Pl), Passau (Pa), and Prague (Pr)

	RVI	Pl versus Pa			Pl versus Pr			Pa versus Pr		
		Estimate (SE)	LCL	UCL	Estimate(SE)	LCL	UCL	Estimate (SE)	LCL	UCL
Intercept		14.15 (4.24)	−12.90	26.80	16.56 (4.59)	−23.26	31.80	2.4 (2.60)	−18.15	12.74
Radiation	1	−0.61 (0.14)	−0.96	−0.33	−0.66 (0.15)	−1.01	−0.35	−0.04 (0.09)	−0.21	0.14
Soil compaction	1	−5.57 (1.77)	−10.20	−2.01	−5.40 (1.85)	−10.45	−1.85	0.16 (1.17)	−2.42	2.33
Way	1	−16.09 (5.79)	−28.07	−5.05	−15.28 (6.54)	−27.95	−3.09	0.81 (5.05)	−8.57	10.65
Woody plants <2 m	1	−38.38 (12.93)	−67.98	−12.76	−26.11 (11.94)	−51.32	0.34	12.35 (9.47)	−4.68	34.45
Woody plants >2 m	1	−13.81 (6.65)	−28.48	−0.88	−27.78 (9.43)	−46.30	−8.34	−13.96 (7.75)	−27.73	2.41
Herbs 1	1	−7.71 (2.52)	−13.43	−2.13	−8.46 (2.48)	−14.42	−2.75	−0.74 (1.85)	−4.54	2.93
Herbs 3	1	−0.22 (2.36)	−5.27	4,592	−9.90 (4.29)	−17.94	−1.11	−9.68 (4.11)	−17.23	−1.15
Herbs 2	1	−6.38 (2.91)	−12.90	−0.67	−12.83 (3.28)	−20.00	−6.30	−6.44 (2.14)	−10.64	−2.08
Temperature	0.51	5.77 (7.85)	−3.44	26.28	9.21 (10.86)	1.91	34.53	3.45 (5.22)	−4.07	17.73
Slope	0.47	0.36 (0.58)	−0.51	2.04	0.78 (0.99)	0.10	3.21	0.42 (0.6)	−0.25	2.03

Note

Estimates and confidence intervals correspond to Pa and Pr in comparison to Pl, and to Pr in comparison with Pa. Most important variables are those with relative variable importance RVI > 0.6.

### 3.3 Microhabitat selection in each region

Results of model averaging of the GLMMs based on abiotic and vegetation parameters as potential predictors are shown in Table 4. Microhabitat selection in the core region was affected only by abiotic parameters. The most important variables found were radiation, slope, soil compaction (RVI = 1), and S‐N aspect (RVI = 0.74), with radiation having a positive effect on the presence/absence of *L. viridis, *and slope, soil compaction, and S‐N aspect having a negative effect. A high proportion of the variance was explained by our model, with the larger part being explained by the random intercept (conditional *R* ^2^ = 0.93; marginal *R* ^2^ = 0.20). The inclusion of random intercepts can enormously improve the explanatory capacity of models, and a high conditional *R* ^2^ value is a very common output in GLMM that intend to find the best set of variables to explain the data (Nakagawa & Schielzeth, 2013) (Supporting Information Appendix S4, Table S4.1 for model selection and model averaging separately for vegetation structure and abiotic parameters).

**Table 4:** Microhabitat selection of green lizards in the core (Plovdiv) and in the periphery (Passau, Prague). Table shows the most important variables (relative variable importance RVI > 0.6) among vegetation structure and abiotic factors resulting from model averaging of selected models (∆AIC < 2)

Variable	Estimate	SE	RVI
Plovdiv			
Intercept	15.3877	7.415	
Radiation	0.5275	0.2727	1
Slope	−3.8056	2.3085	1
Soil compaction	−5.7846	1.4432	1
S‐N aspect	−3.6429	2.9139	0.74
Temperature	−1.406	3.5887	0.24
W‐E aspect	0.1214	0.6582	0.14
Passau			
Intercept	−1.03e03	6.02e−03	
Branches	−2.91e02	2.89e01	1
S‐N aspect	−5.44e01	6.02e03	1
pcnm1	4.48e02	6.02e−03	1
pcnm44	−2.13e+02	4.14e02	1
W‐E aspect	4.97e01	6.02e−03	1
Temperature	6.54e02	6.02e−03	1
pcnm6	−4.91e02	6.02e−03	0.9
pcnm9	−60.13	395.32	0.21
pcnm22	−22.36	1,341.55	0.12
Way	7.664	22.18	0.11
Herbs 3	1.24	96.80	0.11
Bare soil	−6.25	18.25	0.10
pcnm16	−0.39	146.65	0.10
Prague			
Intercept	−4.27	5.08	
pcnm1	−72.84	96.15	1
Herbs 1	4.88	3.79	1
Herbs 2	85.42	54.62	1
Slope	27.15	364.24	0.57
Way	72.81	1,027.02	0.57
Herbs 3	−792.12	14,232.53	0.43
Branches	12.87	378.09	0.22
Bare soil	55.08	1,421.35	0.22

Note

In the core, none of the vegetation parameters was retained in the global model. PCNM: Principal coordinates of neighbor matrices correcting for spatial autocorrelation.

The most important variables affecting microhabitat selection in the periphery‐Pa were a combination of vegetation structure and abiotic parameters: Branches, S‐N aspect, W‐E aspects, and temperature (RVI = 1) *Lacerta viridis* in the periphery‐Pa avoided locations with high coverage of branches and selected places with an eastern and southern aspect where temperatures are higher. The model explained most of the variance, with fixed factors explaining almost half of it (conditional *R* ^2^ = 0.99; marginal *R* ^2^‐marginal = 0.43) (Supporting Information Appendix S4, Table S4.2 for model selection and model averaging separately for vegetation structure and abiotic parameters).

Microhabitat selection in the periphery‐Pr was affected only by vegetation structure variables. *Lacerta viridis* in the periphery‐Pr selected places with low structure principally composed by low vegetation (RVI Herbs2, Herbs1 = 1). Most of the variance in the model was explained by fixed factors (conditional *R* ^2^ = 0.61; marginal *R* ^2^‐marginal = 0.60) with a very small proportion being explained by the random intercept (Supporting Information Appendix S4, Table S4.3 for model selection and model averaging separately for vegetation structure and abiotic parameters).

## 4 DISCUSSION

We hypothesized that the microhabitat niche is smaller at the periphery of the distribution of our study species, *L. viridis, *compared to the core and that there should be a higher preference for specific vegetation structures at the microhabitat scale at the periphery. We further hypothesized that in the core, where availability of suitable habitats does not represent a limiting factor, abiotic parameters will determine microhabitat selection. All hypotheses were met in line with Kühnelt's principle (Kühnelt, 1965), which states that the range of colonizable habitats is wider at the core where environmental conditions are optimal, whereas at the periphery conditions are suboptimal and fewer microhabitats are suitable for the species. The niche of vegetation structure and abiotic parameters was smaller in the periphery and was differentiated from the niche in the core. In the periphery, *L. viridis *compensated for the overall lower suitability of environmental conditions by selecting microhabitats with specific vegetation structures that allow it to take advantage of sufficiently suitable conditions. As expected, only abiotic parameters determined microhabitat selection at the core, whereas at the periphery in Prague, only variables of the vegetation structure influenced microhabitat selection. However, in the periphery in Passau, a combination of abiotic and vegetation structure parameters determined microhabitat selection.

Smaller niche size and niche differentiation in the periphery can be the result of either different thermoregulatory behavior, phenotypic plasticity or local adaptation (genotypic changes) to conditions that lay near the limits of suitability. On the one hand, thermoregulatory behavior can allow individuals at the northern (and upper altitudinal) periphery to meet their thermal requirements by stringent selection of optimal habitats, which therefore often determines the peripheral limits of the distribution of ectotherms (Henle et al., 2010; Huang, Porter, Tu, & Chiou, 2014). In the core region, thermal condition should be more benign, thus allowing ectotherms to reach their thermal requirements in a larger number of different habitats. This is the basic idea behind Kühnelt's principle of regional stenoecy (Kühnelt, 1965) and has been shown qualitatively in various lizard species (Böhme & Rödder, 2014). Furthermore, thermoregulatory behavior might avoid selective pressures to act upon physiological traits and is sometimes regarded as the most plausible mechanism to explain patterns of niche differentiation when data relies on realized niche (Araújo et al., 2013; Bogert, 1949; Grigg & Buckley, 2013; Huey, Hertz, & Sinervo, 2003).

On the other hand, thermoregulatory behavior in lizards is more often found to be determinant near the hot extremes of species’ niches, where individuals avoid heat by retreating into burrows or staying under shadow, compared to near the colder limits of the niche (Muñoz et al., 2014). Moreover, for peripheral populations that are not connected with the distribution range of the species (relict populations), in which immigration from more central populations cannot contribute to population persistence, pressure for adaptation is stronger and therefore phenotypic plasticity and local adaptation (genotypical changes) can be more plausible mechanisms shaping smaller niche size and niche differentiation (Blanquart, Kaltz, Nuismer, & Gandon, 2013; Chevin, Lande, & Mace, 2010; García‐Ramos & Kirkpatrick, 1997). Under this scenario, the selective pressure of environmental conditions can result in adjustments of the thermal physiology, like changes in heat and cooling rates, and critical thermal limits, with the range of selected body temperatures (SBT) at the periphery being different and narrower in comparison with core (Brattstrom, 1968; Castilla, Damme, & Bauwens, 1999; Henle et al., 2010; Huey, 1982). For instance, the STB of the common lizard *Lacerta vivipara* differs between locations, with populations in southern latitudes having a higher STB compared with those located at higher latitudes (Patterson & Davies, 1978; Van Damme, Bauwens, & Verheyen, 1986). The lack of connectedness with the continuous distribution range is indeed the case of the populations in Prague, which are regarded as relicts, have overall small size, and are genetically differentiated from other peripheral (but not relict) populations (Böhme & Moravec, 2011). Additionally, there is evidence in several ectotherm taxa that the expression of the potential phenotypic plasticity of a species is higher near its lower thermal limit, which for several taxa have a strong relation with high latitudes (Chown & Terblanche, 2006; Overgaard, Kristensen, Mitchell, & Hoffmann, 2011).

One possible selective pressure acting upon populations in colder northern peripheral regions can be radiation. Contrary to expectation, radiation had a positive effect on the presence of the lizards in the core area but no effect in the peripheral areas. As a consequence, this variable strongly differentiated microhabitats among regions, being lower in both peripheral regions in comparison with the core. Most importantly, the niches of *L. viridis* in peripheral regions were characterized by lower vegetation height than the niche in the core, where higher temperatures can compensate for increased shading by higher vegetation. Thermal conditions and other limiting factors like daily hours of sunshine (Frör, 1986; Laube & Leppelsack, 2007) presumably do not allow such a compensation at the periphery.

In Passau and Plovdiv, selected microhabitats additionally seem to reflect the response to abiotic parameters shaped also by topography. In Plovdiv, the effects of slope and S‐N aspect were six to ten orders of magnitude stronger than the effect of radiation and were negative. This can be explained by the absence of the lizard in the two rocky hills included among the sites we visited in Plovdiv. In the Passau region, the Danube valley is narrow and is characterized by rocky cliffs, above which the habitat changes dramatically into dense mixed forest and oak forest with high coverage of branches. Despite higher radiation values above the cliffs in comparison with the valley (*z *= −3.501*, p* < 0.01) and the relative abundance of forest edges and clearings with potentially suitable vegetation structures, *L. viridis* seems unable to cope with unfavorable microclimatic conditions in the forest to colonize those areas. Similar observations were made for the Taiwanese lizards *Takydromus hsuehshanensis *(Huang et al., 2014). On the other hand, the rocky open valley has a southeastern aspect, with higher temperatures and suitable microclimate for *L. viridis*. Then, in Passau, it can be more difficult for *L. viridis* to compensate for overall climatic conditions (e.g., lower radiation) by just selecting suitable vegetation structures, because topography confines lizards mostly to the lower part of the valley and they lack accessibility to alternative localities with suitable microclimate.

In all three regions studied other lizard species are also present, *Lacerta agilis* in Passau and Prague, *and Lacerta trilineata* and *Podarcis tauricus* in Plovdiv. Although interspecific interactions, like competition, can have an influence in the niche and microhabitat selection of species, we think that in the regions of our study the possible effect of these interactions, if present, will be very low. Theory predicts that in peripheral populations in higher (colder) latitudes individuals are more limited by climatic conditions, while biotic interactions like predation and competition are more important at low latitudinal peripheries (Cahill et al., 2014; Holt & Barfield, 2009; Price & Kirkpatrick, 2009). In Passau and Prague, *Lacerta agilis* occupies much more humid and covered environments than those inhabited by *L. viridis, *which at this part of its distribution range, as our results showed, tends to occupy drier opener places. Evenmore, in Passau, each species occupies completely different habitats and does not occur synoptically (Waitzmann & Sandmaier, 1990). Nevertheless, an influence of the interaction of both species on the niche of *L. viridis* can be expected in southern regions, where the habitat of both species overlaps (Korsós, 1982), due to the trend of *L. viridis* to inhabit more covered areas toward lower latitudinal regions. However, even in this region, analyses at a finer scale have demonstrated significant niche segregation (Babocsay, 1997; Heltai et al., 2015) that allows the coexistence of both species in the same habitat.

In the core region, the habitats used by *Lacerta trilineata*, *Podarcis tauricus, *and *L. virdis* have an overlap in the driest and least covered portion of the niche of *L. viridis* (Mollov, 2011), which corresponds to the most covered and humid habitats inhabited by the other two species. Therefore, an effect of the interaction with other species on the microhabitat selection of *L. viridis *in this region might be possible but only in a reduced portion of its niche and would have shifted the niche toward the conditions in the periphery if the niche would be indeed suppressed. Analyses at the microhabitat scale in another core region, Hungry also suggest coexistence through niche segregation (Babocsay, 1997). Moreover, the differentiation of habitats between *L. trilineata* and *Podarcis tauricus*, and *L. viridis* becomes stronger toward the southern parts of the distribution range of *L. virids*, like in Greece, where *L. viridis* occupies even more covered habitats (Strijbosch, 2001).

### 4.1 Implications for conservation

Our findings have several implications for the management and conservation of core versus peripheral populations of species. Management measures applied for the protection of peripheral populations of *L. viridis* should address the high specialization degree of the species in these regions, their microhabitat selection and their need to compensate for less suitable climatic conditions. In Prague and in Passau, maintenance of low vegetation in sites where the species already occurs is important for the species’ viability, as it will allow individuals to compensate for low radiation. In Passau, management measures are already installed in the lower parts of the valley (below the cliff; O. Aßmann, pers. comm.). However, we suggest that similar measures should be considered in the upper border of the cliff, in order to increase the potentially suitable area for the species. Also, corridors, for example, along forest tracks or powerlines could facilitate connections between suitable habitats below and above the cliffs. We are not aware that measures for maintaining open vegetation are applied around Prague and would recommend considering them for the long‐term viability of *L. viridis*.

In Plovdiv (Bulgaria), where our core study area was located, it is the diversity of habitats and their vegetation structures that matters most for the species. In the core, abiotic conditions suitable for *L. viridis *are met in a wide range of habitat types, including those with high vegetation and branches coverage. Landscape heterogeneity is altogether known to be important for the viability of many species (Brachet et al., 1999), and in the case of the populations of *L. viridis* in the core it is the presence of habitats with different vegetation structures that could represent the highest benefits. This can be considered, for instance, in Natura 2000 planning or in agri‐environmental measures employed so that they also protect scrubland habitats in the region.

In two of the studied regions, Plovdiv and Prague, the species’ habitat was severely fragmented. Recently, Henle et al. (2016) found that peripheral populations of a related lizard species, *Lacerta agilis*, had a higher specialization degree, lower genetic diversity, and were more sensitive to habitat fragmentation compared to those located in the center. A similar pattern of lower genetic diversity and higher sensitivity to fragmentation caused by the narrower niche is likely to occur also in northern peripheral populations of *L. viridis*. Thus, besides protection of high quality habitats, reestablishing connectivity is an important complementary conservation need.

### 4.2 Limitations and outlook

As in many ecological studies dealing with the quantification of spatial ecological patterns, the risk of local processes influencing the geographical correlation with the parameter under study is always present, and in our study, the inclusion of more regions would have allowed a broader generalization of our results. However, we tried as much as possible to counteract this risk by taking data in less plots per site but increasing the number of sites per region. Most importantly, we defined the spatial scale to which the patterns of niche size are related (Chase & Myers, 2011). To do so, we selected regions that had to fulfill two preconditions closely related to processes that occur at a biogeographical scale: (a) to have contrasting ranges of habitat availability representative of different parts of the distribution range (broad in the core and narrow in the northern peripheries) and (b) to have clearly different climatic regimes. Both premises were fulfilled by all three regions in our study. Local processes due to the particularities of each location, like the topography in Passau and Plovdiv, or the disconnectedness in Prague, are of course still present, but their effects might probably be more related with mechanisms (e.g., local adaptation) acting at a rather local scale, than with differences in niche size and microhabitat selection per sé, which might more strongly respond to a spatial gradient of habitat availability and climatic regimes at a larger spatial scale.

Although our study only includes high latitudinal peripheries of the species’ range and the core and lacks data from other locations along the distribution range of the species, we consider this a valuable input given the many empirical gaps in studying species’ range limits, namely, a detailed analysis of the factors affecting species at the core versus periphery (Sexton, McIntyre, Angert, & Rice, 2009). As a next step, it is important to investigate whether limitations in other regions also lead to changes in niche and microhabitat selection compared to the core. The peripheral regions in our study one a relict (Prague) and the other at the tip of a narrow extension of the distribution range of the species (Passau) might not fully represent the northern periphery. In other northern edges, located at the border of the contiguous distribution range, habitat availability might not be broader and climatic conditions might be as limiting as in Passau and Prague, but the persistence of populations might depend more on immigration than on adaptation to specific conditions. Hence, niche would still be smaller compared to the core but probably less differentiated. On the other hand, in low latitudinal regions, interactions with other lizards’ species might have a more important role in restricting the niche than it does in northern peripheries (Cahill et al., 2014).However, the study of the niche and microhabitat selection of several species must be carefully addressed at the proper spatial scales in order to correctly quantify possible overlaps or segregation among species (Heltai et al., 2015), and its effects in the intraspecific comparison of the niche of populations at peripheries with the core.

Other regions not included in our study that could also represent cold range edges are those located at high altitudes. High altitudinal populations of *L. viridis* are located in the central and southern parts of the species’ range, in the Balkan Peninsula from southern Rumania to northern Anatolia (Pafilis & Maragou, 2013; Schmidtler, 1986; Uhrin et al., 2016). Although this regions are characterized either as subtropical or transitional subtropical‐temperate climatic zones (Nojarov, 2017), it is possible that climatic conditions at high altitudes, as well as an expected narrower range of habitats available, have the same effect on the niche size of *L. viridis* as the conditions in temperate peripheries. This can be specially possible in the Carpathians in south Rumania, where there is a more continental climatic regime with less oceanic and subtropical influence, and where some mountainous populations of *L. viridis* have been reported (Strugariu, 2009). As these regions are surrounded by the contiguous distribution range of the species, and therefore, might strongly depend on immigration, compared with the peripheral regions that we visited, niche differentiation might be lower.

Finally, a higher specialization degree is already known to be linked with a higher sensitivity to habitat fragmentation and climate change at the species level (Henle, Davies, Kleyer, Margules, & Settele, 2004; Lancaster, Dudaniec, Hansson, & Svensson, 2015; Vergara & Armesto, 2009). In the same way, peripheral populations may be more specialized than core populations and be stronger affected by these two processes (Cahill et al., 2014; Hampe & Petit, 2005; Henle et al., 2016). Therefore, the identification of differences in niche and microhabitat selection at fine scales in various locations across the distribution range of single species would significantly improve predictions of species distributions under different scenarios of climate change and habitat fragmentation. This would be enormously valuable to prioritize the application of conservation measures at the population level and at regional and local scales.

## CONFLICT OF INTEREST

None declared.

## AUTHORS CONTRIBUTIONS

AMPR and KH conceived the idea; all authors designed the field surveys and defined the data analysis approach; AMPR conducted the field work, analyzed the data, and wrote the manuscript; all other authors provided manuscript reviews and editorial advice.

## DATA ACCESSIBILITY

Data available from the Dryad Digital Repository: https://doi.org/10.5061/dryad.0bg620m.
