# Supplementary material for: Realized niche and microhabitat selection of the eastern green lizard (Lacerta viridis) at the core and periphery of its distribution range
Source: Ecol Evol. 2018 Oct 31;8(22):11322–36. doi: 10.1002/ece3.4612 (PMC6262924; doi:10.1002/ece3.4612)
Supplement: Supplementary file 1 [file ECE3-8-11322-s001.docx]

**APPENDIX**

**Appendix S1: Correlations and variance inflation factor (VIF)**

**Table S1.1.** Correlations and variance inflation factor (VIF) of continuous variables in the dataset used for comparison of microhabitats among regions (all regions included). Spearman rank correlation and Pearson correlation were applied for vegetation structure and abiotic parameters, respectively.

| **Vegetation structure** | |  |  | |  | |  |  |  |  |  |  |  |
| --- | --- | --- | --- | --- | --- | --- | --- | --- | --- | --- | --- | --- | --- |
|  |  |  | **Spearman Rank Correlation** | | | |  |  |  |  |  |  | **VIF** |
|  | Herbs1 | Herbs2 | Herbs3 | Woody | | Woody | | Dry leaves | Rocks_ | Bare soil | Way | Branches |  |
|  |  |  |  | plants<2m | | plants>2m | |  | trunks |  |  |  |  |
| Herbs1 | 1 |  |  |  | | |  |  |  |  |  |  | 2.555 |
| Herbs2 | -0.504 | 1 |  |  | | |  |  |  |  |  |  | 2.701 |
| Herbs3 | -0.297 | 0.142 | 1 |  | | |  |  |  |  |  |  | 2.125 |
| Woody plants>2m | 0.051 | -0.236 | -0.259 | 1 | | |  |  |  |  |  |  | 1.490 |
| Woody plants>2m | 0.060 | -0.145 | -0.325 | 0.187 | | | 1 |  |  |  |  |  | 1.409 |
| Dry leaves | 0.051 | -0.185 | -0.177 | -0.082 | | | 0.157 | 1 |  |  |  |  | 1.463 |
| Rocks_trunks | -0.117 | 0.082 | 0.045 | -0.192 | | | -0.192 | 0.064 | 1 |  |  |  | 1.281 |
| Bare soil | -0.022 | -0.185 | -0.179 | 0.036 | | | 0.105 | 0.134 | -0.073 | 1 |  |  | 1.283 |
| Way | -0.020 | -0.081 | 0.053 | 0.006 | | | -0.129 | -0.153 | -0.145 | -0.158 | 1 |  | 1.901 |
| Branches | 0.227 | -0.120 | -0.182 | -0.131 | | | 0.229 | 0.209 | -0.057 | 0.136 | 0.107 | 1 | 1.302 |
|  |  |  |  |  | | |  |  |  |  |  |  |  |
| **Abiotic parameters** | |  |  |  | | |  |  |  |  |  |  |  |
|  |  |  | **Pearson Correlation** | | | |  |  |  |  |  |  | **VIF** |
|  | Temperature | Soil | S-N | W-E | | | Slope | Radiation |  |  |  |  |  |
|  |  | compaction | aspect | aspect | | |  |  |  |  |  |  |  |
| Temperature | 1 |  |  |  | | |  |  |  |  |  |  | 1.084 |
| Soil compaction | -0.061 | 1 |  |  | | |  |  |  |  |  |  | 1.226 |
| S-N aspect | 0.087 | -0.075 | 1 |  | | |  |  |  |  |  |  | 1.079 |
| W-E aspect | 0.049 | -0.057 | -0.031 | 1 | | |  |  |  |  |  |  | 1.027 |
| Slope | 0.123 | -0.166 | -0.218 | 0.114 | | | 1 |  |  |  |  |  | 1.131 |
| Radiation | -0.241 | 0.408 | -0.047 | -0.134 | | | -0.186 | 1 |  |  |  |  | 1.302 |

**Table S1.2.** Correlations and variance inflation factor (VIF) of continuous variables in the core Plovdiv. Spearman rank correlation and Pearson correlation were applied for vegetation structure and abiotic parameters, respectively. VIF* are values obtained after removing Herbs 2.

| **VEGETATION STRUCTURE** | | |  |  |  |  |  |  |  |  |  |  |
| --- | --- | --- | --- | --- | --- | --- | --- | --- | --- | --- | --- | --- |
|  |  |  | **Spearman Rank Correlation** | | |  |  |  |  |  | **VIF** | **VIF*** |
|  | Herbs1 | Herbs2 | Herbs3 | Woody | Woody | Dry | Rocks_ | Bare soil | Way | Branches |  |  |
|  |  |  |  | plants<2m | plants>2m | leaves | trunks |  |  |  |  |  |
| Herbs1 | 1 |  |  |  |  |  |  |  |  |  | 14.039 | 1.275 |
| Herbs2 | -0.481 | 1 |  |  |  |  |  |  |  |  | 17.740 |  |
| Herbs3 | -0.238 | -0.109 | 1 |  |  |  |  |  |  |  | 8.525 | 1.347 |
| Woody plants>2m | -0.122 | -0.282 | -0.222 | 1 |  |  |  |  |  |  | 6.721 | 1.212 |
| Woody plants>2m | -0.038 | -0.170 | -0.204 | -0.141 | 1 |  |  |  |  |  | 5.212 | 1.193 |
| Dry leaves | 0.023 | -0.184 | -0.107 | -0.031 | 0.068 | 1 |  |  |  |  | 2.297 | 1.031 |
| Rocks_trunks | -0.066 | -0.089 | -0.073 | 0.008 | -0.111 | -0.056 | 1 |  |  |  | 2.771 | 1.069 |
| Bare soil | -0.071 | -0.176 | -0.180 | 0.207 | -0.019 | -0.078 | -0.056 | 1 |  |  | 3.340 | 1.140 |
| Way | -0.178 | -0.186 | 0.017 | -0.070 | -0.082 | -0.009 | -0.075 | -0.148 | 1 |  | 5.231 | 1.112 |
| Branches | 0.270 | -0.055 | -0.149 | -0.195 | 0.212 | 0.033 | -0.061 | -0.118 | -0.011 | 1 | 1.188 | 1.184 |
|  |  |  |  |  |  |  |  |  |  |  |  |  |
| **ABIOTIC PARAMETERS** | |  |  |  |  |  |  |  |  |  |  |  |
|  |  |  | **Pearson Correlation** | |  |  |  |  |  |  |  | **VIF** |
|  | Temperature | Soil | S-N | W-E | Slope | Radiation |  |  |  |  |  |  |
|  |  | compaction | aspect | aspect |  |  |  |  |  |  |  |  |
| Temperature | 1 |  |  |  |  |  |  |  |  |  |  | 1.057 |
| Soil compaction | 0.130 | 1 |  |  |  |  |  |  |  |  |  | 1.285 |
| S-N aspect | 0.059 | 0.019 | 1 |  |  |  |  |  |  |  |  | 1.121 |
| W-E aspect | 0.057 | 0.077 | -0.103 | 1 |  |  |  |  |  |  |  | 1.040 |
| Slope | -0.024 | -0.001 | -0.264 | -0.053 | 1 |  |  |  |  |  |  | 1.172 |
| Radiation | 0.050 | 0.194 | -0.043 | -0.039 | 0.054 | 1 |  |  |  |  |  | 1.078 |

**Table S1.3.** Correlations and variance inflation factor (VIF) of continuous variables in the periphery Passau. Spearman rank correlation and Pearson correlation were applied for vegetation structure and abiotic parameters, respectively.

| **VEGETATION STRUCTURE** | | |  |  |  |  |  |  |  |  |  |
| --- | --- | --- | --- | --- | --- | --- | --- | --- | --- | --- | --- |
|  |  |  | **Spearman Rank Correlation** | | |  |  |  |  |  | **VIF** |
|  | Herbs1 | Herbs2 | Herbs3 | Woody | Woody | Dry leaves | Rocks_ | Bare soil | Way | Branches |  |
|  |  |  |  | plants<2m | plants>2m |  | trunks |  |  |  |  |
| Herbs1 | 1 |  |  |  |  |  |  |  |  |  | 2.271 |
| Herbs2 | -0.298 | 1 |  |  |  |  |  |  |  |  | 2.491 |
| Herbs3 | -0.127 | 0.015 | 1 |  |  |  |  |  |  |  | 2.125 |
| Woody plants>2m | 0.141 | -0.157 | -0.374 | 1 |  |  |  |  |  |  | 1.218 |
| Woody plants>2m | 0.055 | -0.214 | -0.313 | 0.243 | 1 |  |  |  |  |  | 1.163 |
| Dry leaves | -0.192 | -0.279 | -0.485 | 0.227 | 0.303 | 1 |  |  |  |  | 2.419 |
| Rocks_trunks | -0.320 | -0.073 | 0.080 | -0.215 | -0.180 | 0.041 | 1 |  |  |  | 1.057 |
| Bare soil | 0.029 | -0.048 | 0.106 | 0.056 | -0.044 | -0.130 | -0.185 | 1 |  |  | 1.100 |
| Way | -0.080 | 0.078 | 0.047 | -0.113 | 0.047 | -0.113 | -0.153 | -0.081 | 1 |  | 1.035 |
| Branches | 0.179 | -0.204 | -0.260 | 0.227 | 0.153 | 0.184 | -0.289 | -0.073 | -0.003 | 1 | 1.325 |
|  |  |  |  |  |  |  |  |  |  |  |  |
| **ABIOTIC PARAMETERS** | |  |  |  |  |  |  |  |  |  |  |
|  |  |  | **Pearson Correlation** | |  |  |  |  |  |  | **VIF** |
|  | Temperature | Soil | S-N | W-E | Slope | Radiation |  |  |  |  |  |
|  |  | compaction | aspect | aspect |  |  |  |  |  |  |  |
| Temperature | 1 |  |  |  |  |  |  |  |  |  | 1.414 |
| Soil compaction | 0.131 | 1 |  |  |  |  |  |  |  |  | 1.179 |
| S-N aspect | 0.037 | -0.150 | 1 |  |  |  |  |  |  |  | 1.119 |
| W-E aspect | -0.026 | -0.135 | 0.176 | 1 |  |  |  |  |  |  | 1.250 |
| Slope | 0.151 | -0.064 | -0.144 | 0.285 | 1 |  |  |  |  |  | 1.276 |
| Radiation | -0.224 | -0.091 | -0.025 | -0.102 | -0.168 | 1 |  |  |  |  | 1.536 |

**Table S1.4.** Correlations and variance inflation factor (VIF) of continuous variables in the periphery Prague. Spearman rank correlation and Pearson correlation were applied for vegetation structure and abiotic parameters, respectively.

| **VEGETATION STRUCTURE** | | |  |  | |  | |  |  |  |  |  |  |
| --- | --- | --- | --- | --- | --- | --- | --- | --- | --- | --- | --- | --- | --- |
|  |  |  | **Spearman Rank Correlation** | | | | |  |  |  |  |  | **VIF** |
|  | Herbs1 | Herbs2 | Herbs3 | Woody | | | Woody | Dry leaves | Rocks_ | Bare soil | Way | Branches |  |
|  |  |  |  | plants<2m | | | plants>2m |  | trunks |  |  |  |  |
| Herbs1 | 1 |  |  |  | | |  |  |  |  |  |  | 1.770 |
| Herbs2 | 0.336 | 1 |  |  | | |  |  |  |  |  |  | 1.371 |
| Herbs3 | -0.140 | 0.191 | 1 |  | | |  |  |  |  |  |  | 1.593 |
| Woody plants>2m | -0.040 | -0.056 | -0.396 | 1 | | |  |  |  |  |  |  | 1.237 |
| Woody plants>2m | -0.170 | -0.357 | -0.271 | 0.147 | | | 1 |  |  |  |  |  | 1.405 |
| Dry leaves | -0.290 | -0.491 | -0.399 | 0.103 | | | 0.418 | 1 |  |  |  |  | 2.166 |
| Rocks_trunks | -0.027 | 0.143 | -0.074 | -0.080 | | | -0.073 | -0.151 | 1 |  |  |  | 1.159 |
| Bare soil | -0.164 | -0.281 | -0.052 | -0.047 | | | 0.217 | 0.096 | -0.229 | 1 |  |  | 1.274 |
| Way | -0.090 | 0.078 | 0.083 | 0.082 | | | -0.014 | -0.017 | -0.187 | -0.247 | 1 |  | 1.058 |
| Branches | -0.121 | -0.502 | -0.378 | 0.176 | | | 0.567 | 0.568 | -0.151 | 0.094 | -0.072 | 1 | 1.845 |
|  |  |  |  |  | | |  |  |  |  |  |  |  |
| **ABIOTIC PARAMETERS** | |  |  |  | | |  |  |  |  |  |  |  |
|  |  |  | **Pearson Correlation** | | | |  |  |  |  |  |  | **VIF** |
|  | Temperature | Soil | S-N | | W-E | | Slope | Radiation |  |  |  |  |  |
|  |  | compaction | aspect | | aspect | |  |  |  |  |  |  |  |
| Temperature | 1 |  |  | |  | |  |  |  |  |  |  | 1.107 |
| Soil compaction | 0.053 | 1 |  | |  | |  |  |  |  |  |  | 1.446 |
| S-N aspect | -0.012 | -0.042 | 1 | |  | |  |  |  |  |  |  | 1.081 |
| W-E aspect | -0.056 | -0.166 | -0.075 | | 1 | |  |  |  |  |  |  | 1.559 |
| Slope | 0.154 | -0.032 | -0.518 | | 0.029 | | 1 |  |  |  |  |  | 1.909 |
| Radiation | -0.099 | 0.207 | 0.104 | | 0.059 | | -0.320 | 1 |  |  |  |  | 1.681 |

**Appendix S2 – Correction of spatial autocorrelation of residuals (SACR)**


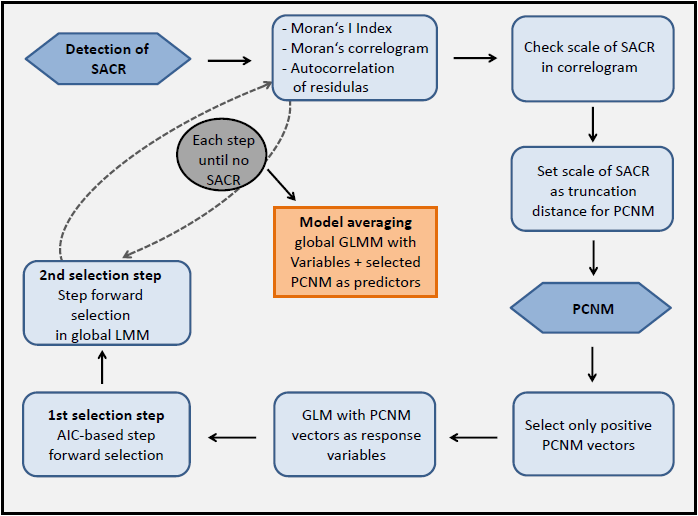
**Figure S2.1.** Process of detection and correction of spatial autocorrelation of model’s residuals (SACR) though principal coordinates of neighbor matrices (PCNM) applied to each global GLMM in the analysis of microhabitat selection. See text for detailed description.

SACR was tested with Moran’s index estimated with the ‘Moran.I’ function in ‘ape’ package of R, Moran’s I correlograms by using ‘correlog’ function in ‘ncf’ package, and autocorrelation of residuals with the ‘acf’ function of the ‘nlme’ package. For the correlogram the function to build lags was set at 100 m and p-values and correlation directions (positive or negative) were checked to estimate at which scale SACR was present. This study focuses on habitat selection at the micro-scale, therefore the presence of SACR was checked at a maximum scale of 1 km. SACR further than 1 km was assumed to be related with ecological processes occurring at broader scales.

The PCNM analysis was performed with the ‘pcnm’ function in the Vegan package. Maximum distance of the lag at which SACR was detected in the correlogram, was set as truncation distance for the PCNM. Only positive PCNM were used in further analysis. Because the PCNM analysis usually generates many eigenvectors (72-81 positive eigenvectors in our models), the number of PCNM to be added into the model as predictors was reduced through a two-steps process (Fig. S2.1). The first step was a forward selection based on AIC for binomial GLMs with the global model containing only all positive PCNM as scope (Ficetola & Padoa-Schioppa, 2009; Sokol *et al.*, 2013). In the second step, the selected PCNM (7 to 10 in our models) were included one by one into the global GLMM containing all other variables (vegetation structure or abiotic parameters), and step forward selected by testing correction of SACR with Moran’s I index, Moran’s correlograms and residuals’ autocorrealtion at each step (Dray *et al.*, 2006; Griffith & Peres-Neto, 2006). Thus, the combination with the smaller number of PCNM that successfully corrected for SACR was finally used for further analysis (Marrot *et al.*, 2015).

For the core area no SACR of model residuals was found in any global model (Table S3.1. Vegetation structure: Moran’s I = 0.28; abiotic parameters: Moran’sI = 0.31; combination: Moran’s I= 0.31).

For peripheral populations in Passau, SACR was found in the global model of vegetation structure (Moran’s I = 1.46e^-07^) at a scale of ~700 m. A total of 81 positive PCNM were obtained, from which 10 were selected in the first step and three (16, 22, 9) in the second step, correcting successfully for SACR (Moran’s I = 0.17). Regarding abiotic parameters in Passau, SACR was also found at a scale of ~700 m (Moran’s I = 1.33e^-15^). A total of 77 PCNM were obtained, from which seven were selected in the first step and four PCNM (22, 44, 6, 1) corrected for SACR in the second step (Moran’s I = 0.68). In both sets, all selected PCNM were found to be important for the microhabitat selection after model averaging (Table S3.2). Therefore, when pulling together variables of both sets, the included PCNM already accounted sufficiently for SACR, and no other PCNM analysis was necessary. Only PCNM 22 of the set of vegetation structure was removed given the model could not converge, but all other PCNM still corrected for SACR (Moran’s I = 0.75).

In the periphery in Prague, SACR was not detected with Moran’s I in any set of variables (vegetation structure: Moran’s I = 0.98; abiotic parameters: Moran’s I = 0.17). However, in the set of abiotic parameters, SACR was detected by the ‘acf’ function and the Moran’s correlogram at a scale of 600 m. A total of 72 PCNM were obtained, and from those eight were selected in the first step and three (72, 42 and 1) in the second (Table S3.3). In the second step selection was based on reduction of SACR tested with the ‘acf’ function and Moran’s correlograms. In the global model combining most important variables of both sets, no SACR was detected.

**References**

Dray, S., Legendre, P. & Peres-Neto, P.R. (2006) Spatial modelling: a comprehensive framework for principal coordinate analysis of neighbour matrices (PCNM). *Ecological Modelling*, **196**, 483-493.

Ficetola, G.F. & Padoa-Schioppa, E. (2009) Human activities alter biogeographical patterns of reptiles on Mediterranean islands. *Global Ecology and Biogeography*, **18**, 214-222.

Griffith, D.A. & Peres-Neto, P.R. (2006) Spatial modeling in ecology: the flexibility of eigenfunction spatial analyses. *Ecology*, **87**, 2603-2613.

Marrot, P., Garant, D. & Charmantier, A. (2015) Spatial autocorrelation in fitness affects the estimation of natural selection in the wild. *Methods in Ecology and Evolution*, **6**, 1474-1483.

Sokol, E.R., Herbold, C.W., Lee, C.K., Cary, S.C. & Barrett, J.E. (2013) Local and regional influences over soil microbial metacommunities in the Transantarctic Mountains. *Ecosphere*, **4**, 1-24.

**Appendix S3: Individual models of vegetation structure and abiotic parameters for the *Comparison of microhabitats among regions***

**Table S3.1.**  Variables selected through model averaging of multinomial models for comparison of microhabitats among regions. Estimates, standard errors (SE) and relative variable importance (RVI) are shown for individual sets of variables: vegetation structure and abiotic parameters. Estimates and SE correspond to Passau (Pa) and Prague (Pr) in comparison with the core Plovdiv.

| Variables | Pa | |  | Pr | |  | RVI |
| --- | --- | --- | --- | --- | --- | --- | --- |
|  | Estimates | SE |  | Estimates | SE |  |  |
|  |  |  |  |  |  |  |  |
| Vegetation structure |  |  |  |  |  |  |  |
|  |  |  |  |  |  |  |  |
| Intercept | 3.78 | 1.14 |  | 5.86 | 1.19 |  |  |
| Way | -8.47 | 3.04 |  | -7.24 | 2.3 |  | 1 |
| Woody plants <2m | -21.31 | 7.4 |  | -11.88 | 3.42 |  | 1 |
| Woody plants >2m | -9.93 | 3.09 |  | -14.09 | 3.7 |  | 1 |
| Herbs 1 | -5.69 | 1.53 |  | -6.11 | 1.43 |  | 1 |
| Herbs 3 | -3.69 | 1.47 |  | -10.14 | 2.69 |  | 1 |
| Herbs 2 | -3.716 | 1.33 |  | -9.46 | 1.92 |  | 1 |
| Rocks_trunks | 1.22 | 2.51 |  | 0.34 | 1.56 |  | 0.26 |
| Branches | -0.77 | 2.03 |  | 0.09 | 0.62 |  | 0.21 |
| Bare soil | -0.98 | 2.58 |  | -0.32 | 1.27 |  | 0.17 |
|  |  |  |  |  |  |  |  |
|  |  |  |  |  |  |  |  |
| Abiotic parameters |  |  |  |  |  |  |  |
|  |  |  |  |  |  |  |  |
| Intercept | -4.46 | 7.27 |  | -14.78 | 8.38 |  |  |
| Radiation | -0.5 | 0.09 |  | -0.53 | 0.1 |  | 1 |
| Slope | 1.09 | 0.5 |  | 1.83 | 0.56 |  | 1 |
| Soil compaction | -3.35 | 1.17 |  | -4.16 | 1.28 |  | 1 |
| Temperature | 5.33 | 5.04 |  | 12.72 | 5.82 |  | 0.75 |
| S-N aspect | -0.21 | 0.48 |  | -0.03 | 0.3 |  | 0.25 |
|  |  |  |  |  |  |  |  |

**Appendix S4: Individual models of vegetation structure and abiotic parameters for the analysis of *Microhabitat selection in each region***

**Table S4.1.** Variables selected in the model averaging of generalized linear mixed model for habitat selection in the core in Plovdiv. Estimates, standard errors (SE) and relative variable importance (RVI) are shown for individual sets of variables: vegetation structure and abiotic parameters.

| Variable | Estimate | SE | RVI |
| --- | --- | --- | --- |
| Vegetation structure |  |  |  |
|  |  |  |  |
| (Intercept) | -3.69633 | 5.78002 |  |
| Rocks_trunks | 1.77245 | 4.56812 | 0.28 |
| Dry leaves | 1.38069 | 4.44620 | 0.21 |
| Herbs 1 | 0.17198 | 0.57128 | 0.17 |
| Woody plants >2m | 0.14448 | 0.77958 | 0.09 |
| Way | 0.08823 | 0.62003 | 0.08 |
| Herbs 3 | 0.04485 | 0.41898 | 0.07 |
| Bare soil | -0.04813 | 0.63451 | 0.07 |
|  |  |  |  |
| Abiotic parameters |  |  |  |
|  |  |  |  |
| (Intercept) | 15.3877 | 7.415 |  |
| Radiation | 0.5275 | 0.2727 | 1 |
| Slope | -3.8056 | 2.3085 | 1 |
| Soil compaction | -5.7846 | 1.4432 | 1 |
| S-N aspect | -3.6429 | 2.9139 | 0.74 |
| Temperature | -1.406 | 3.5887 | 0.24 |
| W-E aspect | 0.1214 | 0.6582 | 0.14 |

**Table S4.2.** Variables selected in the model averaging of generalized linear mixed model for habitat selection in the periphery in Passau. Estimates, standard errors (SE) and relative variable importance (RVI) are shown for individual sets of variables: vegetation structure and abiotic parameters. PCNM: Principal coordinates of neighbor matrices correcting for spatial autocorrelation.

| Variable | Estimate | SE | RVI |
| --- | --- | --- | --- |
| Vegetation structure |  |  |  |
|  |  |  |  |
| (Intercept) | -40.284 | 16.358 |  |
| Bare soil | 33.719 | 26.467 | 1 |
| Branches | -37.81 | 24.41 | 1 |
| pcnm16 | 47.383 | 23.083 | 1 |
| pcnm22 | 238.556 | 104.436 | 1 |
| pcnm9 | -64.229 | 34.589 | 1 |
| Way | 58.026 | 39.074 | 1 |
| Herbs 3 | 11.665 | 6.037 | 1 |
| Herbs 2 | 1.2788 | 2.0666 | 0.28 |
| Herbs 1 | -1.2137 | 3.63 | 0.20 |
| Dry leaves | -0.8665 | 3.71 | 0.15 |
|  |  |  |  |
|  |  |  |  |
| Abiotic parameters |  |  |  |
|  |  |  |  |
| (Intercept) | -2.32E+002 | 3.23E-003 |  |
| pcnm1 | 4.03E+002 | 5.98E+001 | 1 |
| pcnm22 | 3.66E+002 | 6.97E+001 | 1 |
| pcnm44 | -1.12E+002 | 7.31E+002 | 1 |
| pcnm6 | -2.20E+002 | 5.25E+001 | 1 |
| Temperature | 1.27E+002 | 3.13E-003 | 1 |
| S-N aspect | -1.69E+001 | 3.13E-003 | 0.81 |
| W-E aspect | 2.24E+001 | 3.13E-003 | 0.81 |
| Soil compaction | 11.694 | 18.133 | 0.55 |
| Slope | -1.057 | 2.98 | 0.17 |

**Table S4.3.** Variables selected in the model averaging of generalized linear mixed model for habitat selection in the periphery in Prague. Estimates, standard errors (SE) and relative variable importance (RVI) are shown for individual sets of variables: vegetation structure and abiotic parameters. PCNM: Principal coordinates of neighbor matrices correcting for spatial autocorrelation.

| Variable | Estimate | SE | RVI |
| --- | --- | --- | --- |
| Vegetation structure |  |  |  |
|  |  |  |  |
| (Intercept) | -111.23 | 78.38 |  |
| Way | 50.04 | 51.16 | 1 |
| Herbs 1 | 72.74 | 71.43 | 1 |
| Herbs 2 | 1575.42 | 1826.46 | 1 |
| Branches | 20.88 | 19.67 | 0.94 |
| Herbs 3 | -317.15 | 432.45 | 0.84 |
| Woody plants >2m | 28.88 | 39.67 | 0.77 |
| Bare soil | 61.41 | 67.36 | 0.74 |
| Rocks_trunks | 127.38 | 323.9 | 0.51 |
| Woody plants <2m | -288.39 | 507.12 | 0.47 |
| Dry leaves | 01.07.62 | 48.46 | 0.27 |
|  |  |  |  |
|  |  |  |  |
| Abiotic parameters |  |  |  |
|  |  |  |  |
| (Intercept) | -5.2368 | 5.403 |  |
| pcnm1 | -13.626 | 10.2763 | 1 |
| Slope | 1.671 | 0.7394 | 0.9 |
| pcnm72 | -3.70848 | 6.41181 | 0.6 |
| pcnm42 | 7.32409 | 12.17195 | 0.58 |
| Radiation | -0.14635 | 0.18696 | 0.56 |
| Soil comp action | -1.97249 | 2.64025 | 0.53 |
| S-N aspect | 0.07441 | 0.35289 | 0.11 |
| Temperature | 0.8315 | 4.06023 | 0.09 |
| W-E aspect | -0.01027 | 0.13885 | 0.02 |
|  | | | |
